# Interface Kinetics Assisted Barrier Removal in Large Area 2D-WS_2_ Growth to Facilitate Mass Scale Device Production

**DOI:** 10.3390/nano11010220

**Published:** 2021-01-16

**Authors:** Abid, Poonam Sehrawat, Christian M. Julien, Saikh S. Islam

**Affiliations:** 1 Centre for Nanoscience and Nanotechnology, Jamia Millia Islamia (A Central University), New Delhi 110025, India; abid.zak@gmail.com (A.); sehrawatpoonam@gmail.com (P.S.); 2 Institut de Minéralogie, de Physique des Matériaux et de Cosmologie (IMPMC), Sorbonne Université, CNRS-UMR 7590, 4 Place Jussieu, 75252 Paris, France

**Keywords:** chemical, vapor deposition, graphene oxide, transition-metal dichalcogenides, WS_2_

## Abstract

Growth of monolayer WS_2_ of domain size beyond few microns is a challenge even today; and it is still restricted to traditional exfoliation techniques, with no control over the dimension. Here, we present the synthesis of mono- to few layer WS_2_ film of centimeter^2^ size on graphene-oxide (GO) coated Si/SiO_2_ substrate using the chemical vapor deposition CVD technique. Although the individual size of WS_2_ crystallites is found smaller, the joining of grain boundaries due to *sp*^2^-bonded carbon nanostructures (~3–6 nm) in GO to reduced graphene-oxide (RGO) transformed film, facilitates the expansion of domain size in continuous fashion resulting in full coverage of the substrate. Another factor, equally important for expanding the domain boundary, is surface roughness of RGO film. This is confirmed by conducting WS_2_ growth on Si wafer marked with few scratches on polished surface. Interestingly, WS_2_ growth was observed in and around the rough surface irrespective of whether polished or unpolished. More the roughness is, better the yield in crystalline WS_2_ flakes. Raman mapping ascertains the uniform mono-to-few layer growth over the entire substrate, and it is reaffirmed by photoluminescence, AFM and HRTEM. This study may open up a new approach for growth of large area WS_2_ film for device application. We have also demonstrated the potential of the developed film for photodetector application, where the cycling response of the detector is highly repetitive with negligible drift.

## 1. Introduction

Lamellar two-dimensional (2D) WS_2_ has immense potential for electronic applications because of its remarkable properties such as tunable direct bandgap [1,2,3], high photoluminescence intensity [4,5], high emission quantum field [6], attractive spin-orbit coupling [6], substantial exciton/trion binding energies [7,8,9], etc., which give WS_2_ superior leverage over other transition-metal dichalcogenides (TMDCs). Unfortunately, the research on WS_2_ is not matured yet, especially large-scale production of single-crystal monolayers [10,11,12]. Challenges lie in reliable synthesis of atomically thick 2D layers and controlled manipulation of electronic properties [13]. Though mechanical cleaving still remains a relatively facile technique to prepare high quality single to few layered WS_2_, problems such as absence of control over thickness, relatively small lateral size and low yield limit its applications and commercialization [14,15]. The most popular techniques to produce few-layered WS_2_ include intercalation driven exfoliation [16], liquid phase exfoliation [17], physical vapor deposition [18], hydrothermal reaction [19], and heat treatment of W and S containing precursors [20]. However, the domain size of developed WS_2_ via aforementioned methods is generally restricted to a few microns (μm), and the production of WS_2_ monolayer films with desired domain size remains a distant dream. To achieve this, several strategies (e.g., atomic layer deposition [21], pulsed-laser deposition [22,23], metal/metal oxide thin films [24], suitable metal substrates [25], etc.), have been suggested to achieve uniform dispersion of precursors over substrates. Presently, large scale continuous growth of TMDCs is highly complex and expensive, and results in poor quality films [9].

Chemical vapor deposition (CVD) remains a challenge for a long time for growing two-dimensional TMDC materials with high crystallinity, desired thickness, and sufficient domain size [10,12,26,27,28]. For WS_2_ growth, typical synthesis approach involves sulfidation of WO_3_ powders at sufficiently high temperature. The growth conditions such as precursor type, process step, and their manipulations during growth, are found to greatly affect the product quality. Most of the reports on CVD grown WS_2_ solely bank on vapor phase reaction between S and W precursors at suitably high temperatures, where a single furnace is used for both precursors. In such systems, there is no control over the temperature of individual precursor, thereby limiting the parameter space of growth reaction [28]; leading to poor uniformity and repeatability. High melting point of WO_3_ powders and sulfurization rate are found to influence the growth process. Achieving large triangle shaped growth is challenging, because of lower vapor pressure of WO_3_ due to its high melting temperature (1473 °C) [29]. This is a serious problem and severely lowers the partial pressure of WO_3_ vapors [29]. Further, the low vapor pressure of WO_3_ also hampers the availability of W atoms on substrate surface. Thus, enhancing the partial pressure of WO_3_ vapors is a natural approach to obtain large sized WS_2_ flakes [12]. This can be accomplished by reducing the pressure of the furnace. However, this increases the S vapor transfer speed, thereby increasing the sulfurization rate [10]. Fast sulfurization is not desirable for the transport and diffusion of precursor vapors on a substrate and may result in aggregation of precursors at certain locations on the substrate, thereby limiting the growth of large sized WS_2_ crystals [10,30,31]. An efficient way involves a trade-off between the increase in partial pressure of WO_3_ vapors and lower the transfer speed of S vapors.

Recently, many researchers have focused on developing monolayer WS_2_ via CVD. Zhang et al. employed low-pressure chemical vapor deposition (LPCVD) technique to synthesize atomically thin triangle shaped WS_2_ crystals on sapphire substrate having single domain size ~50 μm [32]. An improvement was introduced in the CVD process by Cong et al. to effectively increase the concentration of precursors and furnace pressure [33]. They achieved single-domain growths of ~178 μm [33]. Li et al. introduced further changes in the CVD reaction by employing alkali metal halides as growth promoters [34]. The groups of Zhang [32] and Li [34] reported that due to strong reducing nature, the mixing of suitable amount of H_2_ enhances the sublimation and sulfurization of WO_3_ precursor. Fu et al. [35] investigated the influence of CVD reaction temperature and gas flow rate (comprising a mixture of 97% Ar and 3% H_2_) on the growth size and morphology of WS_2_ films. The optimized growth conditions yielded a domain size of ~52 μm. Rong’s group [28] achieved precise control over S introduction time by utilizing a two-zone furnace and obtained very large area WS_2_ films of 370 μm domain size. Recently, Liu et al. [36] reported sequential synthesis of several one-, and two-dimensional nanomaterials by intentionally creating initial low sulfur conditions, to divide the growth process in two stages: the first stage is a partial reduction and the second one is sulfurization. By this, they could exploit a WO_3-*x*_ intermediate route, and were able to grow large size WS_2_ (150 µm).

TMDCs growth is very susceptible to substrate treatment prior to formation [37]. To assist the nucleation by seeding the substrate with various aromatic molecules, such as -3,4,9,10-tetracarboxylic acid tetrapotassium salt (PTAS), perylene-3,4,9,10-tetracarboxylic dianhydride (PTCDA), and reduced graphene oxide (RGO), perylene encourages the lateral growth of TMDCs. However, this seeding technique helps to grow the large area mono-to few layer TMDCs but continuous film from the abovementioned technique is still limited. Although the CVD method has many advantages, the coordination among various growth parameters is highly complex and requires further elucidation. 

In this work, we present a novel approach for large area synthesis of WS_2_ on graphene oxide (GO) surface via CVD under high temperature conditions of 1070 °C. Upon transformation to RGO during growth process, GO becomes a mixed *sp*^3^-*sp*^2^ bonded network, where nano-sized *sp*^2^-patches of size ~3–6 nm, are randomly distributed with more than 60–70% coverage [38,39,40]. The *sp*^2^-patches are six-fold aromatic carbon atoms with C=C bonds, whereas major percentage of *sp*^3^ bonds constitutes epoxides (C–O) and hydroxyl (−OH) groups in the basal plane and somewhat lesser amount of carboxyl (−COOH) and carbonyl (−C=O) functionalities in the edges [38,39,40]. These *sp*^2^-chemical bonds act as a seed to promote the growth of large area WS_2_ film. The as-developed WS_2_ layers are polycrystalline comprising mainly mono- to few layers. The single-crystal domains are triangular and hexagonal in shape, with sizes up to 60 μm on Si/SiO_2_ and ~15 μm on the GO coated Si/SiO_2_, respectively. The domain size of WS_2_ film on GO coated Si/SiO_2_ is much smaller than that on non-GO substrate, but the development of large continuous surface makes a great footprint in WS_2_ growth, attributed to C=C assisted coalescence of high-density polycrystalline grain boundaries into large domain area. Suitable explanation is given how *sp*^2^-patches comprised of C=C bonds are pivotal in the expansion of domain size of WS_2_ film. Surface roughness of the top layer of substrate is a well-recognized technique for nucleation of nanostructure growth and is cross-checked with intentionally created rough surface on silicon wafer. The proposed device fabrication technique is well suited for mass production of identical devices, and therefore bids for commercial scale development. Moreover, this technique can be extended to the fabrication of other TMDCs. We have further demonstrated the use of the developed film for photodetector applications.

## 2. Materials and Methods

WO_3_ powders (≥99.995% trace metals basis), sulfur (≥99.998% trace metals basis), natural graphite powders (flakes size: 45 μm, ≥99.99% trace metals basis), and sodium nitrate (NaNO_3_, ≥99.995% trace metals basis) were commercially secured from Sigma Aldrich, India. Sulfuric acid (H_2_SO_4_) and potassium permagnate (KMnO_4_) have been supplied by Thermo Fisher Scientific, India and DI water was obtained from Merck (Darmstadt, Germany). For experimental purposes, only analytical grade materials/chemicals were procured and used as received.

The morphology, size and structure of as-synthesized WS_2_ were examined via a transmission electron microscope (TEM) (JEOL JEM F-200, Tokyo, Japan) working at 200 kV. The samples for TEM investigations were kept on the conventional holey carbon Cu grid. High-resolution transmission electron microscopy (HRTEM, JEOL, Tokyo, Japan) studies reveal the uniformity and high quality of WS_2_ samples grown on both Si/SiO_2_ and GO coated Si/SiO_2_ substrates. Basic studies of WS_2_ growth and the domain size were performed on an optical microscope (Leica DM4 P, Wetzlar, Germany). Raman spectrophotometers (WITec Alpha 300RA, Ulm, Germany and LabRAM HR800 HORIBA JY, Kyoto, Japan) fitted with a Peltier cooled CCD detector and confocal microscope were operated for verifying the crystallinity, defects and number of layers. A diode laser of excitation wavelength 532 nm was used to excite the samples. A 100X objective lens with spot size of 1 µm was used to focus the excitation onto the samples. The thickness of grown sample was analyzed by atomic force microscopy (AFM) fitted with the Raman system. Surface morphology of the CVD grown WS_2_ on Si/SiO_2_ and GO coated Si/SiO_2_ was studied on a scanning electron microscope (SEM) (Nova Nano SEM 450, FEI, Oregon, USA). The photoluminescence (PL) measurements were performed by fluorescence spectrometer (Agilent Technologies, Carry Eclipse fluorescence spectrometer, California, USA). For PL investigations, the prepared samples were dispersed in ethanol by simply ultrasonicating the growth substrate for few minutes and isolating the upper one-third solution into a quartz cuvette. 

## 3. Results and Discussion 

Two approaches have been adopted to synthesize monolayer WS_2_ of large domain size, where CVD is employed to grow WS_2_ on two substrates, i.e., Si/SiO_2_ and GO-coated Si/SiO_2_ (Si/SiO_2_/GO), with the aim to extend the domain size of WS_2_ and uniform coverage of the entire substrate area (see Section 3.1 and Section 3.2). 

### 3.1. First Approach: Mono- to Few-Layered WS_2_ Growth on Si/SiO_2_

**Figure 1:**
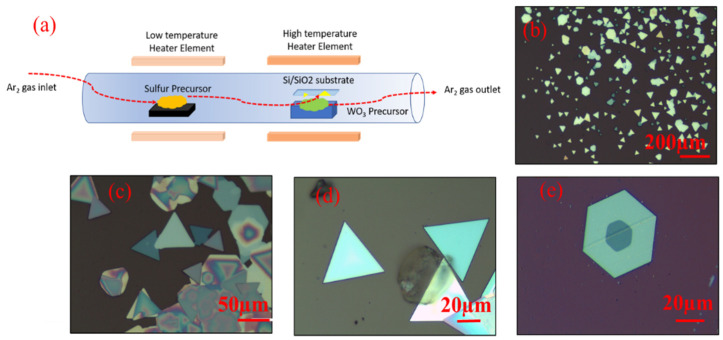
(**a**) Experimental setup. (**b**–**e**) Optical images of synthesized WS_2_ film showing the presence of mono-to-few layered WS_2_ on Si/SiO_2_ substrate.

WS_2_ growth was carried out inside a two-zone tube furnace having separate high- and low-temperature zones, denoted as HT and LT, respectively, as shown in Figure 1a. Ultrahigh purity argon at flow rate of 100 sccm under atmospheric pressure was used as carrier gas to transport sulfur vapors to the HT zone. In a typical experiment, WO_3_ powders (200 mg of pure grade 99.9%) was placed in an alumina crucible at the center of HT-zone of the furnace. Si/SiO_2_ substrate (10 mm × 10 mm) was placed on the alumina crucible in inverted position so as to allow WO_3_ vapor to hits the substrate and deposit on it. The sulfur powder (400 mg of pure grade 99.5%) was loaded upstream, and heated in LT-zone of the furnace. The HT- and LT-zone temperatures were controlled in such a way that the temperature in both the zones simultaneously reach at 1070 °C and 220 °C, respectively [28]. Monolayer WS_2_ grains were grown on Si/SiO_2_ substrate having small domain size and the growth remained scattered. The reaction occurring at the surface of the substrate can be summarized as:

(1) $$2{\mathrm{WO}}_{3}+7S\to 2{\mathrm{WS}}_{2}+3{\mathrm{SO}}_{2}.$$

Thus, the vapor phase reaction between tungsten oxide and sulfur, produces tungsten disulfide and sulfur dioxide as end products. The optimized time duration for WS_2_ growth is found to be 45 min, once the temperature of HT zone reaches 1070 °C. After completing the growth process, the CVD system was allowed to cool naturally to the room temperature.

Figure 1b–e shows the optical images of the WS_2_ sample grown on Si/SiO_2_ substrate. Two types of crystallites are visible: (a) equilateral triangles, and (b) hexagons; both are characteristic of WS_2_ single crystal formation. Most of the crystals include monolayers. However, bi- and tri-layered regions are also visible at the center of large monolayer crystals, which is supposed to be the nucleation site, even for further growth of multi-layers [41]. 

Formation of hexagons in case of multilayer grains originates from the random orientation of individual WS_2_ layers and differences in crystal orientation, where each orientation acts as nucleation site [41,42,43]. The side length of the crystals in case of equilateral triangles is found to be ~60 μm, which is reasonably large. The CVD growth of TMDCs is generally believed to be a self-limiting process [44]. The lateral size of WS_2_ increases with the partial pressure of chalcogen precursors [45], while its thickness is determined by the partial pressure of transition metal precursors [46]. We have kept the weight of sulfur powders to be the twice of the weight WO_3_ (chalcogen precursor). Thus, the lateral growth dominates in our case and could explain the growth of large size single crystals [46]. Furthermore, we could find WS_2_ single crystals in variable sizes, ranging from 100 nm to 60 μm on the same Si/SiO_2_ substrate. The growth duration is found to directly influence the area of grown crystals, before the crystal growth reaches its limit. Therefore, large sized WS_2_ crystals can be formed if the growth time is kept sufficiently long.

- Electron Microscope Analysis

**Figure 2:**
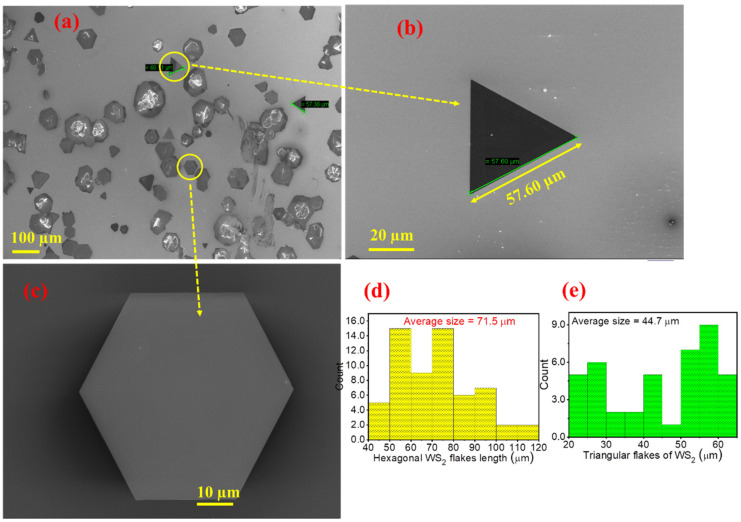
(**a**–**c**) FESEM micrographs of the WS_2_ layers grown on Si/SiO_2_ substrate at various magnification levels and (**d**,**e**) Size distribution histogram of hexagonal and triangle WS_2_ flakes.

Figure 2a–c shows the FESEM images of WS_2_ samples deposited on Si/SiO_2_ substrate, corroborating the findings of optical microscopy, indicating both equilateral triangles and hexagons. In case of WS_2_, the growth of equilateral triangles suggests single crystallinity; whereas the hexagonal or star-like structures result from several rotationally symmetric grains [4,9,26,46,47]. The size distribution histogram of hexagonal and triangle flakes of WS_2_ is shown in Figure 2d–e. Where the average size of the hexagonal and triangular flake is found to be 71.5 μm and 44.5 μm, respectively. The ratio of the hexagon to the triangular flakes over the substrate is 2:1. Notably, the growth products, though consisting of monolayers, are scattered over the substrate and do not cover the entire substrate surface. 

**Figure 3:**
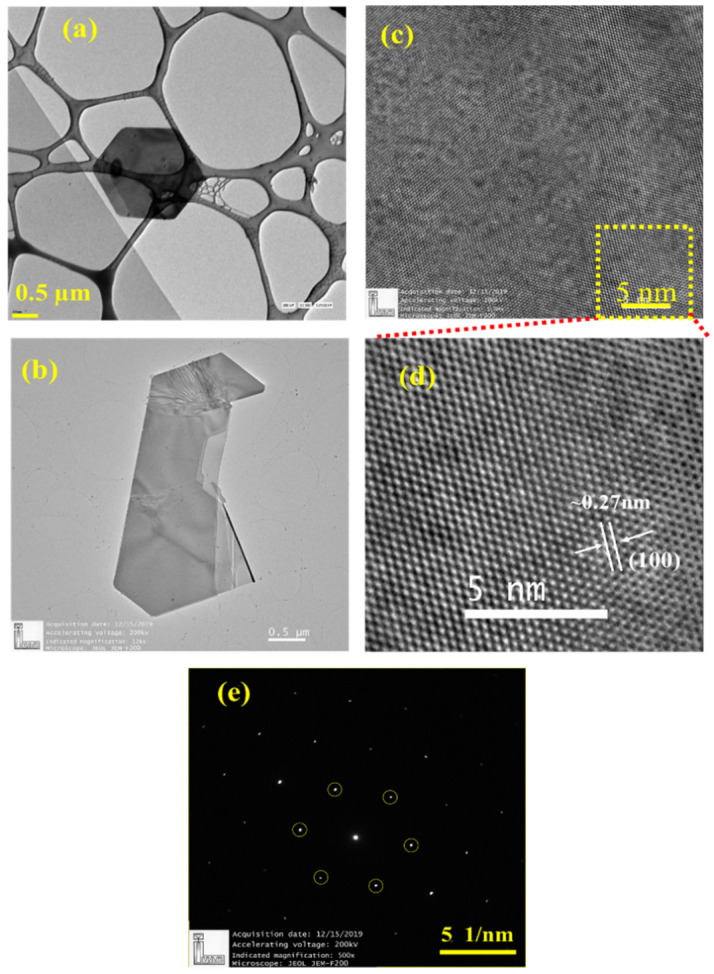
The TEM images of WS_2_ flakes shown in (**a**,**b**) both at low magnification, (**c**) at high magnification. (**d**) zoomed view of (**c**), and (**e**) SAED pattern of the zoomed area.

Figure 3a–c shows a low magnification bright field TEM imaging of WS_2_ crystals suspended on a holey carbon Cu grid. For TEM imaging, first the WS_2_ grown onto the Si/SiO_2_ substrate was ultrasonicated in ethanol for 20 min to get the WS_2_ flakes. After this, the suspension was centrifuged at a speed of 3000 rpm for 15 min to remove unwanted contamination or to allow settling down of heavy particles. Finally, top one-third supernatant was used for TEM analysis. We used HRTEM and selected area electron diffraction (SAED) to characterize the WS_2_ domains. Figure 3a shows the hexagonal flakes with lateral dimension of about ~2.5 µm. In Figure 3b, the distorted cum truncated WS_2_ triangle is clearly observable. At higher resolutions (Figure 3c,d), the crystalline nature of the flakes is clearly displayed. In Figure 3e, the selected area electron diffraction (SAED) pattern is indicated, which further confirms the highly crystalline morphology of as-grown WS_2_.

- Raman Analysis

**Figure 4:**
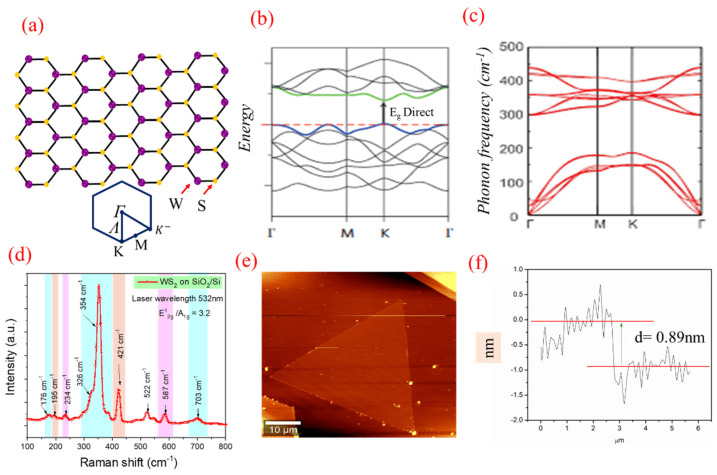
(**a**) Real space lattice structure and Brillouin zone in reciprocal space, (**b**) electronic band structure (*E-K* diagram), (**c**) phonon dispersion, (**d**) Raman spectrum, (**e**) AFM image of WS_2_ monolayer crystal grown on Si/SiO_2_ substrate and (**f**) AFM cross-section height profile for the deposited sample revealing a thickness of the grown film to be ~0.89 nm.

**Figure 5:**
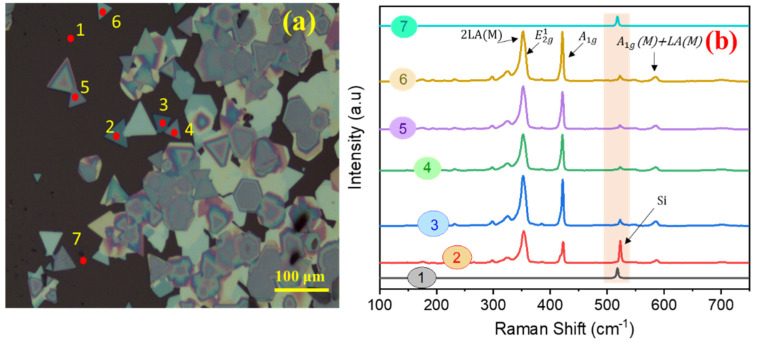
(**a**) Optical microscope image of WS_2_ deposited over Si/SiO_2_, indicating the selected measurement spots, and (**b**) Raman spectra of the spots identified in (**a**) where peak at 520 cm^−1^ in all the spectra is the LO mode of the silicon wafer.

The *E-k* diagram and the phonon dispersion in Brillouin zone for WS_2_ are shown in Figure 4a–c. Raman spectroscopy was employed to detect both the in-plane (*E*_2g_^1^) and out-of-plane (*A*_1g_) phonon modes. Figure 4d shows Raman spectrum of the WS_2_ flakes deposited over the Si/SiO_2_ substrate, indicating a strong peak at 354 cm^−1^ (2LA(*M*)), signifying a longitudinal acoustic mode at *M* point. This signals the monolayer growth of WS_2_ [9,26]. The peak at 421 cm^−1^ corresponding to the *A*_1g_ mode, although weak, is also observed. As is widely reported, the separation between these two peaks increases from 67 to 72 cm^−1^ with increasing the number of layers from single- to tri-layer due to a red-shifting of 2LA(*M*) mode [26,48,49,50,51]. However, the observed peak separation of ~67 cm^−1^ is the fingerprint of WS_2_ monolayer growth. Its topography, revealing a triangular shape probed via Atomic Force Microscopy (AFM), is shown in Figure 4e,f. The whole crystal is almost atomically flat and clean. The height profile in Figure 4f indicates the as-grown WS_2_ on Si/SiO_2_ having a step size of 0.89 nm; reaffirming the growth of a WS_2_ monolayer. To verify the uniformity in the flakes, Raman mapping studies were performed from one edge to the other (Figure 5a,b). The synthesized WS_2_ flakes were further analyzed by recording multiple Raman spectra across the area of post WS_2_ grown substrate. Typically, seven measurement sites were selected on the top surface, and it can be concluded that we have achieved the monolayered WS_2_ flakes. As seen in Figure 5a,b, the spots 1 and 7 only show the Raman-active LO mode of Si, while spots 2, 3, 4, 5, and 6 express the Raman features of WS_2_ monolayers, and interestingly, the Raman result is also found supplemented by AFM studies.

- Photoluminescence (PL) Analysis

**Figure 6:**
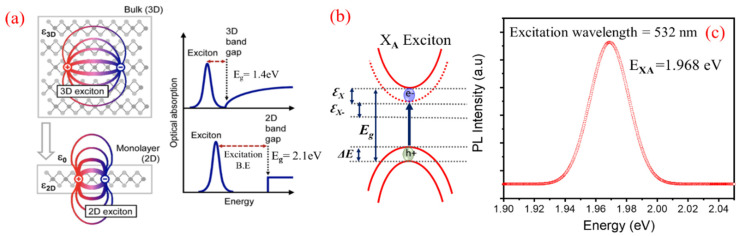
(**a**) Schematic diagram showing the reduced dielectric screening on the e-h pair bonding in a typical 2D material [38,39,40]. (**b**) Neutral A-excitonic transition (*X*_A_) in the energy level diagram, and (**c**) PL spectra of WS_2_ grown over Si/SiO_2_ substrate.

The PL properties of 2D-TMDCs are mainly governed by their excitonic features [1,7,8,9,52]. In the limiting case of 2D materials, as shown in Figure 6a, the lines of electric field between the hole and electron start extending beyond the material, thereby reducing the dielectric screening and enhancing coulomb interactions [36,38,39]. A strong coulombic force is the main reason behind strong photoemission efficiency. This enhanced coulomb interaction suggests formation of bound e-h pairs or “excitons”, having greater binding energies making them stable even at room temperature [7,52,53,54]. In case of monolayer 2D material, significant deviation is expected from the hydrogenic model, prevalent to describe Wannier-excitons in inorganic semiconducting materials [7,51]. Therefore, the calculation of binding energy of excitons in case of single-layered WS_2_ needs to consider modified potential [54]. High intense peak is observed at 630 nm (1.968 eV) in the as-synthesized monolayer WS_2_, which mainly originates from neutral-exciton (*X*_A_) emission due to direct transition between the lowest conduction band (CB) and the highest valence band (VB) at the same *K*-point in the Brillouin zone [7,49,55,56,57,58], and schematically represented in Figure 4c. The fundamental bandgap of a single layer WS_2_ is ~2.1 eV, but the large excitonic binding energy of 0.032 eV renders the optical bandgap measured at 1.968 eV [8,50,51,52] as shown schematically in Figure 6a,c shows the PL spectra of WS_2_ flakes on the Si/SiO_2_ substrate. Figure 6b represents the Neutral A-excitonic transition (XA) in the energy level diagram. The FWHM values of the PL spectra lie between 25 to 35 meV for both the approaches which is in accordance with the literature and further establish the fact of monolayer formation [59,60]. The presence of enhanced coulombic interaction at higher distant points suggests more likelihood of formation of higher-order excitons, i.e., trions and bi-excitons [51].

### 3.2. Second Approach: Uniform Growth of WS_2_ Flakes on Spin-Coated GO

**Figure 7:**
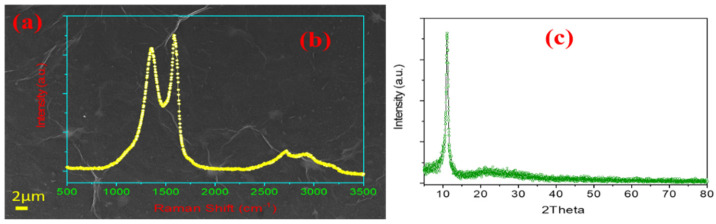
(**a**) FESEM micrograph, (**b**) Raman spectrum, and (**c**) XRD pattern of the GO film deposited on Si/SiO_2_ substrate before the growth of the WS_2_ layer.

In this approach, we have spin coated pre-synthesized graphene oxide (GO) solution over the Si/SiO_2_ substrate prior to CVD growth. Hummers’ method [38,39,40] has emerged as a well-established technique to prepare graphite oxide which can then be ultrasonicated to yield graphene oxide (GO). Briefly, natural graphite powders (5.0 g), H_2_SO_4_ (120 mL) and NaNO_3_ (2.5 g) were mixed via continuous stirring. During stirring, the temperature was kept below 10 °C in order to check overheating of the mixture. After obtaining a uniform mixing, KMnO_4_ (15.0 g) was added to this mixture by stirring for 24 h at room temperature. A condensed light brown colored slurry was obtained, which was diluted with deionized water (150 mL) and stirring for 2 h at 98 °C. The resulting mixture was vigorously washed with HCl and deionized water, to yield graphite oxide. Subsequently, graphite oxide was ultrasonicated (37 kHz, 500 W) for 3 h. The resulting suspension was centrifuged at 5000 rpm and GO was obtained by collecting the top one-third supernatant from the centrifugation product. A suspension of GO was prepared by mixing 5 mg GO in deionized water via ultrasonication. The substrate for WS_2_ growth was prepared by spin coating (2500 rpm) 100 μL GO suspension on a Si/SiO_2_ wafer. Figure 7a includes the SEM micrograph of the GO film coated over Si/SiO_2_ substrate. The GO flakes appear uniformly dispersed over the surface of the substrate, and are expected to act as a seed layer for the growth of WS_2_ film. In Figure 7b, the Raman spectrum of the GO film is displayed in 500–3500 cm^−1^ range, in which the D-, G- and 2D-band are clearly defined at 1354, 1592, and 2712 cm^−1^, respectively. Further, XRD analysis was performed to confirm the quality of prepared GO. The XRD pattern in Figure 7c shows the peak at 2θ ≈ 11°, which validates the high quality of the GO flakes.

**Figure 8:**
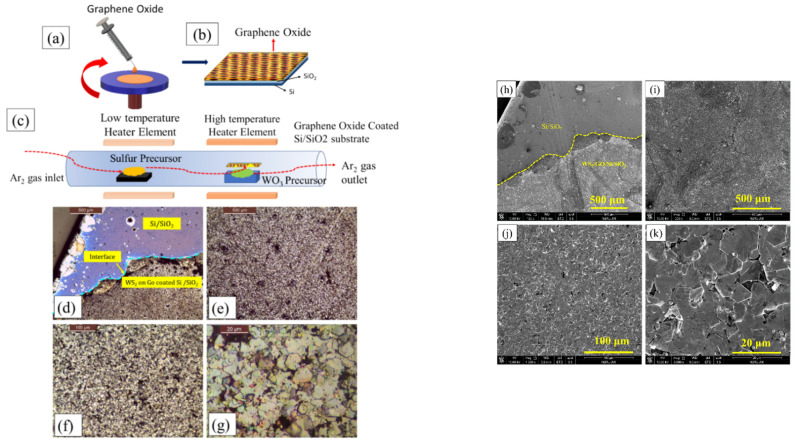
(**a**) Spin coating GO solution over Si/SiO_2_ substrate, (**b**) GO coated Si/SiO_2_ substrate, (**c**) CVD experimental setup used for WS_2_ growth, and (**d**–**g**) optical microscope images of WS_2_ grown on GO coated Si/SiO_2_ substrate, and (**h**–**k**) FESEM micrographs of WS_2_ grown over GO coated Si/SiO_2_ substrate.

**Figure 9:**
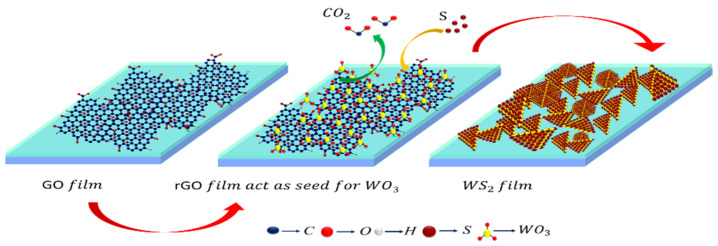
Schematic diagram to represent WS_2_ growth mechanism on GO coated Si/SiO_2_ substrate.

The WS_2_ growth was performed similar to that described in Section 3.1 (i.e., WS_2_ grown at Si/SiO_2_ substrate). The only difference is the coating of Si/SiO_2_ substrate with GO, which is expected to get converted into RGO due to in-situ heating encountered in the HT-zone during the growth process. The synthesis process is schematically illustrated in Figure 8a–c. Figure 8d–g shows the Optical images of the WS_2_ grown on the GO coated Si/SiO_2_, where in Figure 8d clear interface is shown between WS_2_ film and the Si substrate. Figure 8h–k shows the FESEM micrographs at different magnification. In this approach, sufficient amount of sulfur vapor is taken into consideration for the reaction, similar to the first approach. Here, it is noteworthy to say that the *sp*^2^-carbon nanostructures in the RGO matrix fulfill three-pronged objectives: (i) they improve the surface adhesion, thereby enhancing local adsorption of WO_3_ molecules; (ii) they reduce WO_3_ to yield WO_3-*x*_; and finally, (iii) they also act as active sites for heterogeneous nucleation, as demonstrated in Figure 9.

Upon heating, WO_3_ vapors reach the surface of the substrate. A portion of these vapors diffuses and reacts with *sp*^2^-bonded carbon patches of RGO, resulting in the formation of an intermediate phase, WO_3-*x*_, along with the release of CO_2_. Thus, an increase in the concentration of WO_3_ vapors, results in simultaneous increase in the CO_2_ and a corresponding decrease in the size of *sp*^2^-carbon patches. Further, at elevated temperatures, WO_3-*x*_ molecules gain the tendency to migrate and aggregate around the *sp*^2^-bonded carbon patches. These carbon patches or ‘carbon nanostructures’ are expected to act as heterogeneous nucleation sites; where nucleation takes place via sulfurization of adjoining WO_3-*x*_ molecules. The synthesis kinetics involve heterogeneous and homogeneous nucleation reactions, occurring simultaneously and competing with each other, as the main growth processes [61,62]. Initially, due to a lower surface free-energy barrier, heterogeneous nucleation outpaces the homogeneous nucleation [59]. In addition, the local high concentration of WO_3-*x*_ facilitates the formation of bilayer nuclei. In case of perfectly overlapping bilayer formation, the nucleation shifts from the bottom layer to the second layer almost instantly. If growth does not stop at this point, few-or multi-layer WS_2_ flakes are formed. Nonetheless, a complete understanding of growth mechanics warrants further investigations. The growth process can be summarized in the following chemical reactions [61,62]:

WO_3_ +(*x*/2)C → WO_3−x_ + (*x*/2)CO_2_, (2)

WO_3_ + (*x*/2)S → WO_3−x_ + (*x*/2)SO_2_, (3)

WO_3−x_ + (7 − *x*)/2S → WS_2_ + (3 − *x*)/2SO_2_. (4)

The use of GO produces substantially different growth characteristics than the isolated islands observed during the first approach. The WS_2_ islands now coalesce to form nearly continuous films on centimeter scale as is evident in the optical images and SEM micrograph (Figure 8). Apart from the monolayers, multilayered growth is also visible. The multilayered growth is more favored at either the nucleation sites or grain boundaries (the locations shown by pointing arrows). Continuous monoto-few layer growths on centimeter scale are representative of the samples grown via the second approach, where the Si/SiO_2_ substrate is coated with GO. We have found the size of the individual WS_2_ crystals less than 15 μm, which is much larger than those reported for h-BN substrates, where a domain size of less than 1 μm is observed [63,64,65]. Although it is smaller than the WS_2_ grown on pristine Si/SiO_2_ substrate, its continuous expansion on the entire substrate has strong merits and fulfils the objectives of this work, as well as the requisite need for device applications.

**Figure 10:**
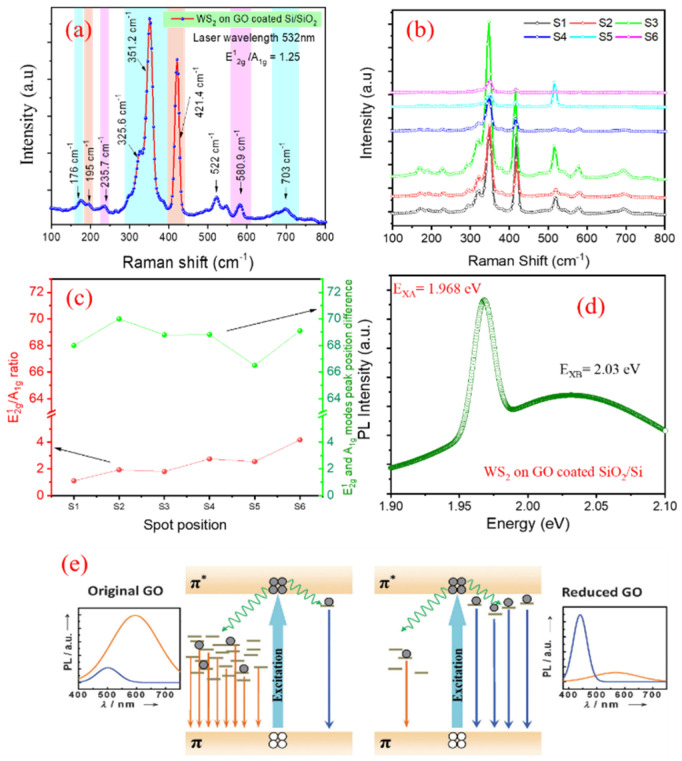
(**a**) Raman spectrum and (**b**) Raman spectra as a function of spot on different positions (**c**) Intensity ratio and difference of the peak as function of spot (**d**) Photoluminescence (PL) of WS_2_ grown on GO coated Si/SiO_2_ substrate, and (**e**) Evolution of PL spectra due to the transformation of sp^3^- to *sp*^2^-bonded region during the reduction of GO to RGO. Copyright Abid, Sehrawat, Islam, Mishra, Ahmad. Under a Creative Commons Attribution 4.0 International License. Reproduced with permission from [38]. Copyright 2012 Wiley.

Figure 10a shows the Raman spectrum of a WS_2_ film grown on GO coated substrate. The peaks present at 351.2 and 421.4 cm^−1^ is relatable with the $E_{2g}^{1}$ and *A*_1g_ Raman modes. Besides, the $E_{2g}^{1}$ and *A*_1g_ modes are found to be slightly red- and blue-shifted, respectively. Our concern is to study the shift in $E_{2g}^{1}$ and *A*_1g_ peaks due to thickness variation in a mixed multi-layered WS_2_ flake. The increase in number of layers strongly enhances the out-of-plane vibrations, while coulomb interactions tend to decrease the frequency of in-plane vibrations, thereby causing monotonous increase in frequency separation between $E_{2g}^{1}$ and *A*_1g_ peaks [58]. While there is little variation in peak positions, small modifications in the relative intensities between $E_{2g}^{1}$ and *A*_1g_ modes are clearly observed. Samples synthesized via first approach have a slightly higher *E*_2g_^1^ intensity, with the ratio $E_{2g}^{1}$/*A*_1g_ of ~3.2. Conversely, the *A*_1g_ intensity is higher for the second approach, where $E_{2g}^{1}$/*A*_1g_ ratio is of ~1.25. Moderate changes in the Raman intensity ratio are believed to indicate changes in the number of layers for exfoliated WS_2_ [46,66]. We have performed Raman mapping analysis at multiple spots of the same sample to confirm the uniformity of WS_2_ across the substrate area as shown in Figure 10b,c. It is evident that there is slight variation in the separation as well as intensity ratio of in-plane and out-plane vibrational peaks as a function of sample spot location; and a summary of the peak position, difference in Raman peak frequency and intensity ratio of the peaks is tabulated in Table 1. The shift in the frequency of 2LA (M) and A_1g_ modes has been employed as a measure of the number of layers. However, this approach to determine the number of layers is erroneous due to the close proximity of 2LA (M) and $E_{2g}^{1}$ phonon modes. It has been found that with increasing the number of layers, the intensity ratio (*I*_2LA(M)_/*I*_A1g_) decreases from 2.2 (for single layer) to 0.47 (for bulk) [64]. At the first instance, it looks the possible growth of mono- to few-layered WS_2_. However, in case of monolayer MoS_2_, such small intensity variations in *E*_2g_^1^ and *A*_1g_ peaks were correlated to the differences in electronic doping levels or strain as WS_2_ and RGO effectively combine to form heterostructures [67]. Generally, TMDCs are highly prone to significant number of chalcogenide vacancies [59,64,65]. Sulphur vacancies exist in WS_2_, thus introducing localized donor states deep inside the bandgap, which can be subsequently filled by environmental impurities to make it doped. As of now, various competitive approaches exist to find out the origin of anomaly in the interpretation of peak intensity ratio and shift in peak position of Raman modes. Nonetheless, the contribution to the intensity changes due to sulfur content in WS_2_ films as a dopant, and local strain in between WS_2_ and RGO, cannot be ignored [66].

**Figure 11:**
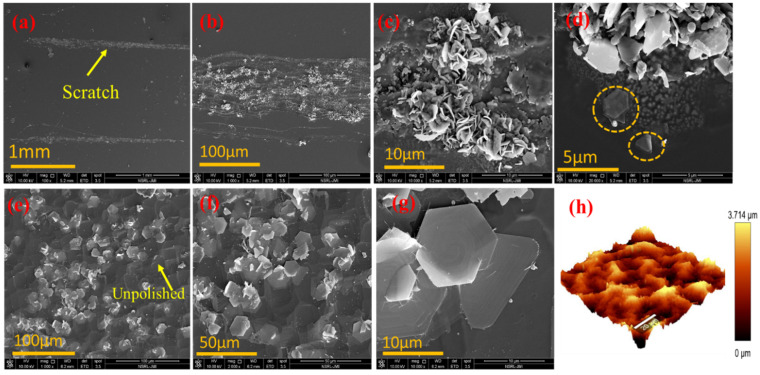
FESEM micrographs of WS_2_ grown on scratch marks made on polished surface of Si wafer (**a**–**d**) and WS_2_ grown on unpolished Si wafer (**e**–**g**). In both cases, other CVD growth parameters were constant. (**h**) The optical profilometer image (surface microscopy) of the GO coated sample surface before WS_2_ deposition. The scale bar indicates the surface roughness of the sample close to 3.72 µm.

The PL spectra, shown in Figure 10d, comprise with neutral A-exciton (*X*_A_) at 1.968 eV as also observed in case of WS_2_ growth on Si/SiO_2_ substrate. A striking difference observed in WS_2_ growth on GO-coated Si/SiO_2_ substrate, is the appearance of a low intensity peak at 2.03 eV (611 nm), attributable to the weak transition of carriers from disorder induced states within π-π^*^ band structure of RGO [67] (schematically shown in Figure 10e). Our laser excitation visible wavelength does not cover the intense emission peak around 440 nm. A comparative study in terms of PL spectral positions, FWHM and shift in peak positions are put in Table 2. Surface roughness, in particular, the sharp edges, is reported to help in catalyst-free CNT growth [68]. To ascertain the role of surface roughness on the continuous growth of WS_2_, we conducted CVD growth in identical conditions: first, on polished surface, marked with scratches on polished side of silicon wafer; and second, on backside, the unpolished surface of the same wafer. In the polished wafer side, WS_2_ debris having no shape and sizes of flakes, are observed on and around the scratch marks. SEM images (Figure 11a–d) show few vertically aligned and flower petal shaped flakes with some horizontal flakes on the scratch marked region having negligible presence of triangular as well as hexagonal WS_2_ crystallites.

In contrast, we have achieved horizontally grown WS_2_ crystallites, majority in hexagonal shape on the unpolished surface (Figure 11e–g). In both cases, thickness of WS_2_ flake(s) is multilayered. It may be inferred that surface roughness does also play an important role in the WS_2_ growth. The optical profilometer image (surface microscopy) of the GO coated sample surface before WS_2_ deposition is shown in Figure 11h. It is to be noted that surface of RGO film is highly rough in nature; and this may be one of the sources of nucleation sites too, in addition to the major contribution from high density *sp*^2^-bonded patches, to enhance the joining of grain boundaries, resulting in continuous WS_2_ film growth over GO coated Si/SiO_2_ substrate. Therefore, it may be understood that continuous growth is possible in GO coated substrate, in which *sp*^2^-patches of carbon purely act as a seed or catalyst that enhance growth origins.

The present work has shown the successful growth of mono to few layer WS_2_ film of square-centimeter size and triangular shape on graphene-oxide-coated Si/SiO_2_ substrate using CVD technique. The real issue addressed in this work is the large area coverage of the WS_2_ continuous film; however, many of the researchers have also tried to resolve this issue by using various approaches such as atomic layer deposition, pulse laser deposition, and metal/metal oxide thin film or noble metal substrates, etc. Although these strategies yield large area continuous TMDCs, these techniques also suffer from high costs, increased complexity, and sometimes poor quality. Further, to be useful, WS_2_ grown over inert metal needs etching via acidic or basic (e.g., concentrated HF, KOH and NaOH) solutions, before transferring to Si or flexible substrates. Usually, the transfer processes not only end up in damaging the grown material (WS_2_), but produce substrate residues and negative environment impact, as well. We successfully grew the continuous WS_2_ film at very low cost by simply coating the Si/SiO_2_ substrate with graphene oxide (GO) and this technique is highly reproducible. Table 3 enlists the optical properties of WS_2_ nanoflakes deposited on different substrates. However, HRTEM and Raman spectroscopy confirm the high crystallinity and quality of the grown WS_2_ flakes. Note that the Raman peak frequencies difference (66–72 cm^−1^) and the PL peak position (2.00–1.92 eV) as well as intensity fluctuates over the WS_2_ triangles as enlisted in Table 2. The change of frequency and/or width of Raman modes of 2H-type transition-metal dichalcogenides can be used to evaluate the sample quality because they are strongly sensitive to external electrostatic doping (*A_1g_* mode) and lattice strain (*E*_2g_^1^ mode) [69,70]. Wang et al. found that *E*_2g_^1^ mode exhibits obvious red-shift when increasing strain [70]. In contrast, Chakraborty demonstrated that a softening and broadening of the *A*_1*g*_ mode occur with electron doping, whereas the *E*_2g_^1^ mode remains essentially inert [69].

- Photoconductive response of sensor prepared with GO coated Si/SiO_2_

**Figure 12:**
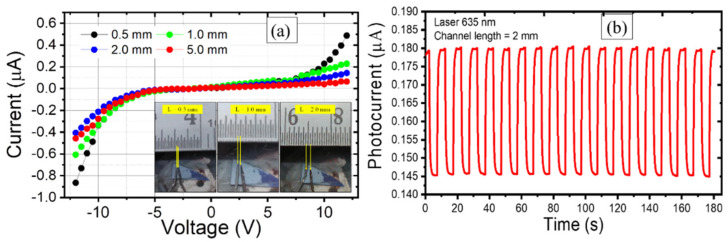
(**a**) Current vs. voltage characteristics at variable channel length and (**b**) photoconductive response under laser wavelength 635 nm.

To check the continuity of the grown film, I-V characteristics were monitored in the voltage range of −12 V to +12 V using Keithley SCS 4200 for variable channel length (from 0.5 to 5.0 mm). The inset of Figure 12a shows the digital photographs of the samples, where the electrical probes are set at different channel lengths. The observed I-V curves indicate that flakes grown over the GO modified substrate are interconnecting and form a continuous network. Figure 12b demonstrates the possible use of developed film as photodetector where the change in photocurrent under illumination of laser wavelength of 635 nm, keeping the laser power constant at 40 mW, is shown. The cyclic response is highly reproducible with negligible drift. The photoresponsivity and specific detectivity are found to be 0.9 μA W^−1^ and 0.15 × 10^5^ jones.

## 4. Conclusions

Efforts have been made to understand the growth mechanics of large domain size of mono-to-few layer growth of WS_2_. Nature of substrate is utilized to understand the intricate mechanism behind continuous film growth. Large area (domain size of around 60 μm), mono-to-few layer WS_2_ crystals of triangular shape, were grown on pristine Si/SiO_2_ and GO-coated Si/SiO_2_ substrates, using CVD technique with a two-zone furnace. For GO coated Si/SiO_2_ substrate, uniform mono-to-few layer growth of WS_2_ crystals is achieved, although the size of WS_2_ crystals is smaller than those grown on pristine Si/SiO_2_ substrate. In case of growth on Si/SiO_2_ substrate, monolayer WS_2_ grain boundaries are in isolation, and highly scattered on the substrate surface. Further tests were conducted to examine the role of surface roughness, as reported in the past for catalyst-free CNT growth on pristine silicon surface. We conducted CVD growth in identical conditions, one on polished surface, marked with scratches; and the other one, on the unpolished surface of the same wafer. Strikingly, WS_2_ growth exclusively on rough surface in both the cases, confirmed that surface roughness does play an important role. In addition, high density *sp*^2^-bonded patches enhance the joining of grain boundaries, to enable continuous WS_2_ film growth over GO coated Si/SiO_2_ substrate. It is concluded that the combination of high-temperature CVD with reduced graphene oxide surface is supposed to be a favorable condition for TMDCs growth with full coverage of the substrate. The idea of GO coated Si/SiO_2_ substrate may be employed for mass production of identical devices, and therefore bids for commercial scale development and can be extended to other TMDCs. 

## Figures and Tables

**Table 1:** Summary of Raman studies of WS_2_ nanoflakes deposited on different spot of GO coated substrate.

Sample	Spot	Raman Peak Frequency (cm^−1^)		Frequency Difference	Intensity Ratio
		*E*_2*g*_	*A*_1*g*_		
WS_2_/SiO_2_/Si	1	354.3	421.1	66.7	1.75
	2	349.7	418.7	69	1.15
	3	350.1	419.2	69.1	0.96
	4	350.1	419.4	69.3	0.99
	5	352.4	419.3	66.9	1.2
	6	-	-	-	-
WS_2_/GO/SiO_2_/Si	S1	350.8	418.8	68.0	1.10
	S2	351.0	421.2	70.2	1.93
	S3	349.7	418.25	68.5	1.80
	S4	350.1	419.0	68.9	2.75
	S5	352.9	419.6	66.5	2.55
	S6	351.0	~420.1	69.1	4.19

**Table 2:** Summary of Photoluminescence (PL) studies of WS_2_ nanoflakes deposited on different substrates.

Sample	Energy (eV)	FWHM (meV)	
	*E_XA_*	*E_XA_*	*E_XB_*
WS_2_/Si/SiO_2_	1.968	25.3	-
WS_2_/GO/SiO_2_/Si	1.968	35.5	35.6

**Table 3:** Summary of optical properties of WS_2_ nanoflakes deposited on different substrates.

Sample	Raman Peak Frequency (cm^−1^)		A-Exciton Energy (eV)	Ref.
	*E*_2*g*_	*A*_1*g*_		
WS_2_/Al_2_O_3_	352.7	421.2	2.000	[10]
WS_2_/SiO_2_/Si	353.0	418.3	1.920	[18]
WS_2_/Au	356.2	420.9	1.935	[25]
WS_2_/SiO_2_/Si	352.5	419.0	1.977	[12]
WS_2_/sapphire	354.0	412.0	2.000	[26]
WS_2_/SiO_2_/Si	~350	~416	1.949	[28]
WS_2_/SiO_2_/Si	354.0	421.0	1.968	this work
WS_2_/GO/SiO_2_/Si	351.2	421.4	1.968	this work

## Data Availability

The data presented in this study are available on request from the corresponding author.

## Appendix A

*Photoresponsivity (R_λ_)*

The ratio of photocurrent to the incident intensity of the laser wavelength λ is defined as photoresponsivity of the photodetector and it can be calculated as [71]:

(A1) $$R_{\lambda }=\frac{\Delta I_{ph}}{P\cdot A}.$$

*Photosensitivity (S)*

It is defined as the ratio of change in current under illumination and dark current [72]:

(A2) $$S\;\left(\%\right)=\frac{I_{photo}-I_{Dark}}{I_{Dark}}\times 100.$$

*Specific Detectivity*

It represents the ability of the detector to capture the weakest signal, which can be mathematically expressed as [71]:

(A3) $$D=\frac{R_{\lambda }\sqrt{A}}{\sqrt{2eI_{Dark}}},$$

where *A* represents the effective area of photodetector, and *e* is the electronic charge.
